# Air bronchogram integrated lung ultrasound score to monitor community-acquired pneumonia in a pilot pediatric population

**DOI:** 10.1007/s40477-020-00547-7

**Published:** 2021-01-06

**Authors:** Riccardo Inchingolo, Roberto Copetti, Andrea Smargiassi, Rafael Emanuele Gerardi, Emanuele Giovanni Conte, Giuseppe Maria Corbo, Antonio Gatto, Chiara Pierandrei, Lavinia Capossela, Ilaria Lazzareschi, Piero Valentini, Libertario Demi

**Affiliations:** 1 Pulmonary Medicine Unit, UOC Pneumologia, Fondazione Policlinico Universitario A. Gemelli IRCCS, Largo Gemelli, 8, 00168 Rome, Italy; 2 Emergency Department, University Hospital Cattinara, Trieste, Italy; 3 Pulmonary Medicine Unit, Ospedale “C. E G. Mazzoni”, Ascoli Piceno, Italy; 4 UOC Pneumologia, Università Cattolica del Sacro Cuore, Roma, Italy; 5 UOC Pediatria, Fondazione Policlinico Universitario A. Gemelli IRCCS, Rome, Italy; 6 School of Medicine and Surgery, Università Cattolica Sacro Cuore, Rome, Italy; 7 UOC Pediatria, Università Cattolica del Sacro Cuore, Rome, Italy; 8 Ultrasound Laboratory Trento, Department of Information Engineering and Computer Science, University of Trento, Trento, Italy

**Keywords:** Air bronchogram, Children, Imaging, Score, Ultrasound, Pneumonia

## Abstract

**Aims:**

Chest ultrasound is a non-invasive method for evaluating children with suspected community-acquired pneumonia (CAP). We evaluated the prognostic role of change of ultrasonographic (US) air bronchogram in management of CAP in terms of: rate of complicated CAP, change of empiric antibiotic therapy, relationship to defervescence time, and length of hospitalization.

**Methods:**

Patients with CAP and radiographic evidence of lung consolidation were prospectively enrolled. Chest US examinations were performed within 12 h from admission and after 48 h. A new grading system (USINCHILD score) based on presence and features of air bronchogram was adopted.

**Results:**

Thirty six patients were stratified into two groups according to the presence of an increase of at least 1 grade of US score (Δ US grade), expression of an improvement of lung consolidation. Δ US grade after 48 h ≥ 1 was associated with an increased risk of complicated CAP (*p* value 0.027) and a longer defervescence time (*p* value 0.036). Moreover, Δ US grade ≥ 1 was predictive of a short hospitalization (*p* value 0.008).

**Conclusions:**

USINCHILD score could be an innovative biotechnology tool for the management of pediatric CAP.

**Trial registration number and date of registration:**

NCT03556488, June 14, 2018.

**Graphic abstract:**

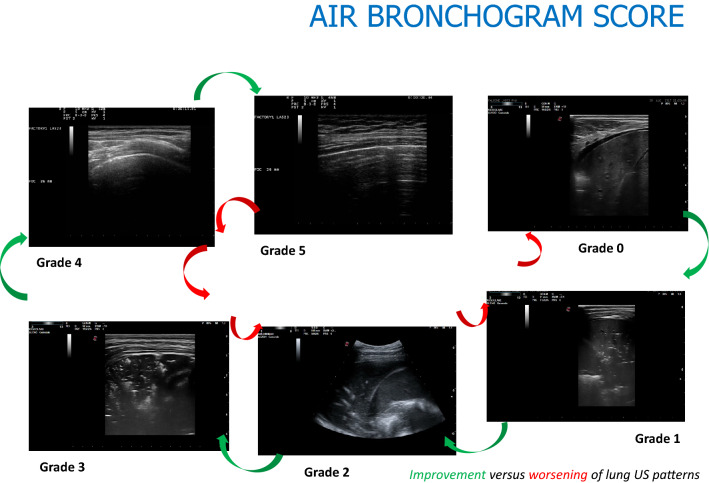

**Electronic supplementary material:**

The online version of this article (10.1007/s40477-020-00547-7) contains supplementary material, which is available to authorized users.

## Introduction

Pneumonia is a leading cause of childhood morbidity worldwide [1]. Each year, 2 million children younger than 5 year die of pneumonia, representing 20% of all deaths in children within this age group [2]. Although difficult to quantify, it is believed that up to 155 million cases of pneumonia occur in children every year worldwide [2]. In the developed world, the annual incidence of pneumonia is 3–4 cases per 100 children, 5 years old [3, 4].

According to the British Thoracic Society (BTS) guidelines, clinical symptoms and signs are usually sufficient to diagnose community-acquired pneumonia (CAP) in children [8]. Thus, the BTS guidelines do not recommend performing chest radiograph (CXR) in all children with typical pneumonia signs and symptoms. On the other hand, recent BTS Paediatric Pneumonia Audit revealed that in many children suspected of CAP, chest radiograph was done to confirm the diagnosis. Moreover, subsequent follow-up radiographs were commonly overused, causing unnecessary radiation exposure [9].

Many studies showed that ultrasound (US) findings of lung and pleural diseases described in adults are also found in pediatric patients [7–11] and that lung US is a good clinical tool to detect consolidation in children affected by pneumonia [12–15]. A recent meta-analysis of these studies highlights the attractive diagnostic role of US for pediatric CAP [16].

In 2009, Lichtenstein and co-workers showed that in patients with alveolar consolidation displaying air bronchograms on ultrasound, the dynamic air bronchogram indicated pneumonia, distinguishing it from resorptive atelectasis [17]. Air bronchograms are punctiform or linear hyperechoic artifacts within the consolidation. Dynamic air bronchogram is the term used for the centrifugal inspiratory dynamic of air bronchograms, ultrasonographic finding usually detected in lung consolidation due to pneumonia. Conversely, static air bronchograms are found in resorptive atelectasis [17].

Previous studies explored if LUS could be useful to diagnose and monitor the response to treatment of pneumonias in adults [18, 19].

In 2013, Caiulo et al. showed LUS is not inferior to CXR in identifying pleuro-pulmonary alterations in children with suspected pneumonia. During the course of the disease, the lack of ultrasonographic improvement was related to the lack of clinical recovery [20]. Bouhemad et al. showed that LUS can evaluate the size of the consolidated area and the improvement in lung aeration [21]. More recently, Musolino et al. suggested that a deep and fixed air bronchogram in the initial LUS might be able to predict the development of complicated pneumonia [22].

Lung US follow-up allows to verify complications, to avoid additional radiation exposures, and to monitor the evolution of the disease [11, 31].

We evaluated the prognostic role of the change of air bronchogram, a typical ultrasonographic finding of lung consolidation due to pneumonia, in the management of CAP in terms of: (1) impact on rate of complications; (2) change of antibiotic therapy not guided by microbiological examinations; (3) relationship to the time of resolution of clinical signs; (4) length of hospitalization.

## Materials and methods

### Study subjects

This was a pilot single-center observational prospective study performed at Fondazione Policlinico Universitario A. Gemelli IRCCS in Rome, Italy. Ethical approval for the study was obtained [13528/ID:2001]. Children with age from 1 to 18 years, with suspected CAP [5] and radiographic evidence of lung consolidation, admitted to Paediatric Unit, were consecutively enrolled. A written informed consent was given from parents. Children with history of gestational age < 36 weeks, a previous diagnosis of cystic fibrosis or congenital pulmonary disease, were excluded.

### Study design

The current primary outcomes were: the rate of uncomplicated CAP; the time to resolution of fever, the change of antibiotic therapy not guided by microbiological examinations, and, finally, the length of hospitalization.

The lack of previously published studies exploring the same US score led us to plan an estimated enrollment of at least 30 patients in 1 year.

### Methods

At admission, the pediatric evaluated clinical signs of respiratory distress [i.e., tachypnea, dyspnea, retractions (suprasternal, intercostals, or subcostal), grunting, and nasal flaring, apnea], presence of altered mental status, pulse oximetry values ≤ 90% on room air and temperature. Furthermore, microbiologic tests (nasopharyngeal swab specimens, [NPS] and pneumococcal urinary antigen) to detect causative pathogens of CAP, and laboratory tests (complete blood cell count, acute-phase reactants, and C-reactive protein [CRP]) were performed. Finally, if chest radiography (CXR) was performed before the admission to Paediatric Unit, the radiological exam was not repeated.

US evaluation was performed by Pulmonologists using MyLab Alpha (Esaote S.p.a., Rome, Italy) machine with convex (3.5–5 MHz) and linear (7.5–12 MHz) probes based on the size of the chest. We did not adopt the eight-zone scanning technique usually suggested in emergency setting [32]. Conversely, we explored all chest regions that can be scanned implementing the growing belief that ultrasonographic insonation is a pillar of bedside physical examination [33, 34]. The first examination was performed within 12 h from admission. The Pulmonologists could identify signs of respiratory distress and/or low values of SpO_2_, if present, but they were blinded to clinical findings such as heart rate, blood pressure, and completely blind to radiological and biohumoral findings.

To characterize the lung consolidation from ultrasonographic point of view, a new grading system (USINCHILD score) based on the presence and the features of air bronchogram was adopted.

This score assigns a grade according to these features. The grade 0 indicates a lung consolidation without air bronchogram (video 0); the grade 1 a lung consolidation with static air bronchogram (video 1); the grade 2 a lung consolidation with dynamic air bronchogram (video 2); the grade 3 a lung consolidation with both dynamic air bronchogram and areas of lung recruitment (video 3); the grade 4 a focal sonographic interstitial syndrome (video 4); finally, the grade 5 characterized by normal pleural–parenchymal pattern, expression of resolution of lung consolidation (video 5).

**Fig. 1:**
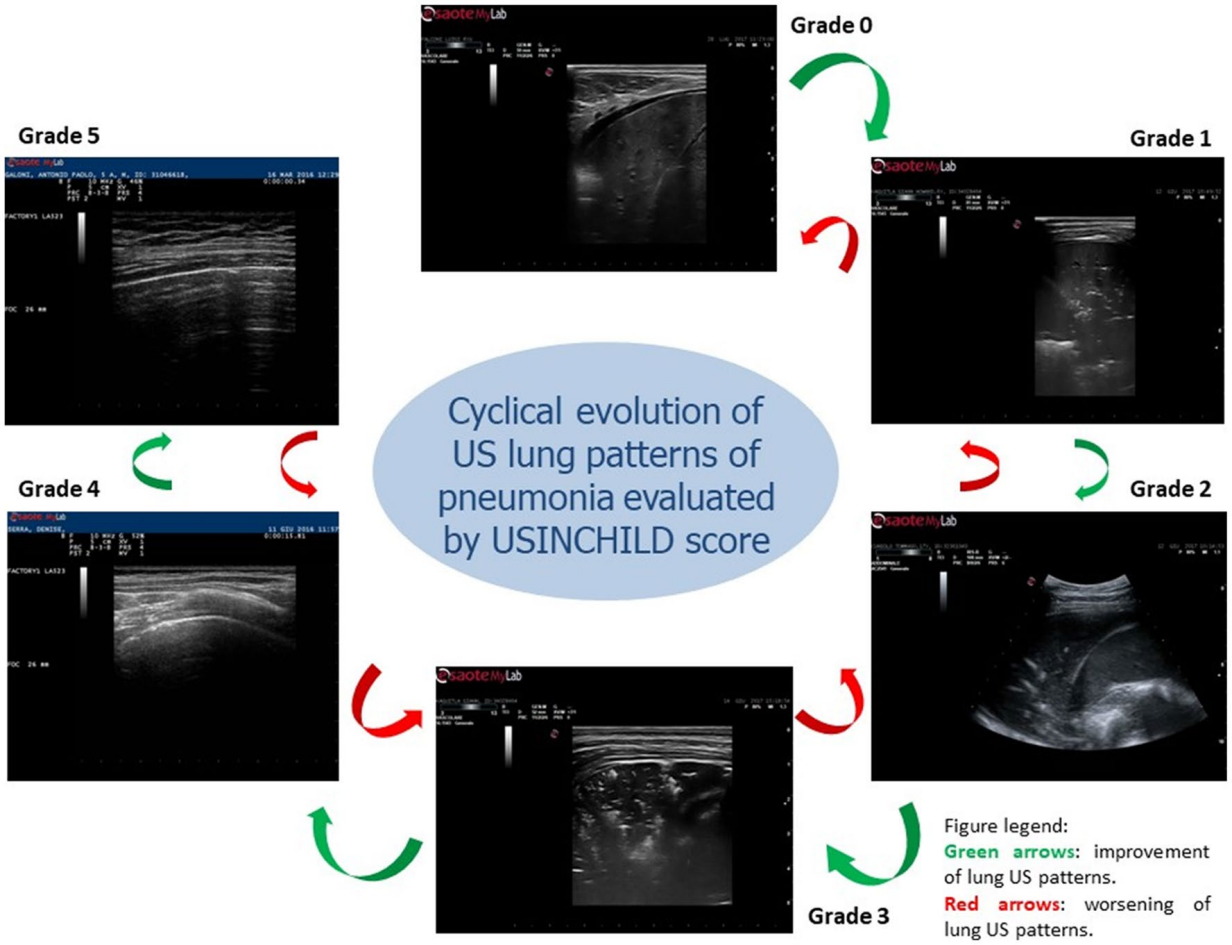
Cyclical evolution of US lung patterns of pneumonia evaluated by USINCHILD score

We adopted a score with the lowest values expression of a greater compromise of lung parenchyma due to inflammation/infection with no evidence of air bronchogram (grade 0) or with only static air bronchogram (grade 1), expression of air trapped in the parenchyma affected by pneumonia. Conversely, the highest values of the score describe the resolution of consolidative process with evidence of vertical artifacts originating from the pleural line—usually described as sonographic interstitial syndrome (video 4), until the normal ultrasonographic pattern is restored (video 5) (Fig. 1).

The air bronchogram described in grade 2 is usually arborescent, an important feature for the scoring definition, previously defined as a specific sign for both community- and ventilator-acquired pneumonia [21, 23, 26].

The features of the grade 3 include a lung consolidation with both dynamic air bronchogram and areas of lung recruitment. Moreover, the identification of this grade requires the presence of the shred sign—a static sonographic sign observed in lung consolidation due to the deeper border of consolidated lung tissue, in contact with the aerated lung, usually shredded and irregular [21, 30].

The grade 4, named sonographic interstitial syndrome, described a pneumogenic pattern of sonographic features of B lines (unusual septal disposition of B lines, blurred, uneven, coalescent B lines and white lung, non-modulated B lines, pseudo B lines) and pleural line (irregular with reduced pleural sliding) in an area previously affected by a consolidation process [35].

The Pulmonologists (RI, AS, REG, and EGC), with at least 5 years' experience in ultrasound collected and stored images and videos (10 s). All US images and clips were reviewed by two Pulmonologists of the Research Group (RI and AS) and by an expert Clinician in chest US (RC), blind to other data to evaluate inter-observational variability by Kappa statistic.

In case of clinical deterioration, children underwent further US evaluations according to pediatric indications.

The identification of complicated pneumonia was based on the presence of: (1) severe respiratory failure requiring assisted ventilation, (2) septicaemia, (3) pleural effusions and empyema, and 4) necrotising pneumonias.

### Analysis

The outcome measures were the rate of complicated CAP at 48 h, the change of empiric antibiotic therapy at 48 h, the defervescence time expressed in hours, and, finally, the length of hospitalization expressed in days.

Descriptive data were reported as mean values and standard deviation (SD) for continuous variables (age, WBC, CRP, time to resolution of fever, and length of hospitalization), whereas frequency of clinical findings, rate of complicated CAP, and rate of upgrade of empirical antibiotic therapy were reported as percentages. Finally, also US air bronchogram grades (at admission and after 48hours) and change of the grade after 48 h [Δ US grade] were reported as percentages.

Comparisons (at admission and after 48 h) were made by Sign test for clinical findings and Wilcoxon signed-rank test for Δ US.

After the stratification of US grades in a post hoc analysis, we identified a priori two groups of patients according to the presence of an increase of at least 1 grade of US score, a plausible clinical expression of an ultrasonographic improvement of lung consolidation.

The first outcome, the association of the change of air bronchogram with the rate of complicated respiratory infections, was tested with Fisher’s exact test. Then, an unweighted logistic regression analysis was performed to evaluate the association between the evolution of the CAP (complicated/uncomplicated) and the presence of an increase of at least 1 grade of US score after 48 h (US grade Δ ≥ 1).

Moreover, Fisher’s exact test was also used to test the correlation between the upgrade of empiric antibiotic therapy and change of USINCHILD score.

Instead, to assess the association between the change of the therapy and several variables (US grade Δ ≥ 1, the change of WBC and CRP after 48 h), we performed a logistic regression analysis.

Subsequently, a Receiver-Operating Characteristic (ROC) curve analysis was performed to test sensitivity/specificity of variations of USINCHILD score and dichotomic variables (complicated CAP and upgrade of antibiotic therapy).

The association between the time to resolution of fever and the presence of an increase of at least 1 grade of US score after 48 h (US grade Δ ≥ 1) was tested by Cox regression. Finally, this test was also used to evaluate the relationship between the days of hospitalization and the change of USINCHILD score. Correlation analysis were also adjusted for sex and age.

STATA version 11 was used for data analysis, data management, and graphics.

## Results

From May to December 2018, we collected data from all patients who met the inclusion criteria and for whom all US images and clips—at admission and after 48 h—were considered of high quality by the Research Team and the expert Reviewer.

Study population was composed by 36 patients (18 females) with average age of 5 ± 4 years.

Demographic and clinical findings of enrolled patients are shown in Table 1.

**Table 1:** Demographics and clinical findings

Patients	[No.]	36		
Female	[No., % of sample]	18 (50)		
Age	[years, M^1^, SD^2^]	5 (±4)		
		At admission	After 48h	
WBC^3^	[M, SD, x10^9^/L]	13.8 (±7.3)	9.2 (±3.8)	
CRP^4^	[M, SD, mg/L]	92.4 (±116.3)	45.1 (±53.9)	
	[No., % of sample]	At admission	After 48h	*p* value^5^
Cough		22 (61)	4 (11)	0.0001
Dyspnea		17 (47)	5 (13)	0.0002
Respiratory Failure		7 (19)	2 (5)	0.0313
Fever		33 (91)	12 (33)	0.0001
Gastrointestinal symptoms		8 (22)	3 (8)	NS
Malaise		21 (58)	9 (25)	0.0002
Sputum		13 (36)	4 (11)	0.0020
Outcomes				
Complicated CAP		No. (% of sample)	9 (25)	
Upgrade of empiric antibiotic therapy		No. (% of sample)	17 (47)	
Time to resolution of fever		Hours (mean, SD)	89 (±62)	
Length of hospitalization		Days (mean, SD)	11 (±7)	

^1^Mean value

^2^Standard deviation

^3^White blood cells

^4^C reactive protein

^5^Sign test

Nine patients (25%) had a complicated CAP and 17 patients (47%) underwent an upgrade of empiric antibiotic therapy. Mean values of time to resolution of fever and length of hospitalization were 89 ± 62 h and 11 ± 7 days, respectively.

The inter-observer agreement (Kappa statistic) of USINCHILD score was 0.85 as concerns the evaluation performed at admission, and 0.89 as concerns the evaluation performed after 48 h.

The frequency distribution of both USINCHILD score at admission and after 48 h and the change of USINCHILD score after 48 h (ΔUS) are reported in Table 2. The change of USINCHILD score after 48 h was statistically significant (Wilcoxon signed-rank test, p value: 0.0001).

**Table 2:** Frequency distribution of USINCHILD score

Grade^*^	At admission	After 48h	Δ US^¥^
	Frequency (% of sample)		*p* value^‡^
0	4 (11)	1 (2)	0.0001
1	15 (41)	7 (19)	
2	4 (11)	5 (13)	
3	9 (25)	5 (13)	
4	4 (11)	13 (36)	
5	0	5 (13)	
Frequency distribution of Δ US^¥^			
Value	Frequency (% of sample)		
− 1	1 (2)		
0	12 (33)		
1	8 (22)		
2	10 (27)		
3	4 (11)		
4	1 (2)		

*0: absent

^1^Static

^2^Dynamic

^3^Dynamic with areas of lung recruitment

^4^Focal interstitial sonographic syndrome

^5^Resolution of consolidation

^¥^Δ US: difference between USINCHILD score after 48hours and at admission

^‡^Wilcoxon signed rank test

Moreover, the patients were dichotomized into two groups according to the presence of an increase of at least 1 grade of USINCHILD score (ΔUS grade) and expression of an improvement of lung consolidation.

The first outcome was the association of the change of USINCHILD score with the rate of complicated respiratory infections (Table 3). Patients with evidence of improvement of USINCHILD score (ΔUS grade ≥ 1) showed a low rate of complicated CAP (Fisher’s exact test, *p* value: 0.0001). Moreover, the evidence of ΔUS grade ≥ 1 was associated with a statistically significant increase of risk of complicated CAP (*p *value: 0.027). Finally, the Table 3 shows the sensitivity and specificity to identify complicated/uncomplicated CAP for each degree of USINCHILD change after 48 h (area under ROC curve: 0.87).

**Table 3:** The association of USINCHILD score with the rate of complicated respiratory infections

Comparison between rate of complicated CAP and change of USINCHILD score				
	US Grade ∆^¥^ < 1	US Grade ∆^¥^ ≥ 1		*p* value
Uncomplicated CAP	5	22	27	0.0001
Complicated CAP	8	1	9	
Tot	13	23	36	
Logistic regression of rate of complicated CAP				
Variable	Odds ratio	Standard error	95% conf. interval	*p* value
US Grade ∆^¥^ ≥ 1	0.0038	0.0095	(0.0000–0.5259)	0.027
∆ WBC **	1.0216	0.1148	(0.8197–1.2734)	0.849
∆ CRP ***	0.9841	0.0094	(0.9659–1.0027)	0.093
Sex	3.2793	4.7661	(0.1900–56.6128)	0.414
Age	1.3290	0.2346	(0.9403–1.8784)	0.107

ROC curve analysis		
US grade Δ^¥^	Specificity	Sensitivity
− 1	0	0.1111
0	0.1852	0.8889
1	0.4815	0.8889
2	0.8519	0.8889
3	0.963	1
4	1	1

^¥^US Grade ∆: difference between USINCHILD score after 48hours and at admission

*Fisher's exact test.

**∆WBC: the difference between WBC evaluated after 48h of follow–up and WBC at admission

***∆CRP: the difference between CRP evaluated after 48h of follow–up and CRP at admission

Table 4 shows the correlation between the change of USINCHILD score and the upgrade of antibiotic therapy not guided by microbiological examinations (Fisher’s exact test, *p* value 0.0467).

**Table 4:** The association of the change of USINCHILD score with the upgrade of antibiotic therapy

Comparison between upgrade of antibiotic therapy and change of USINCHILD score						
Not upgrade Ab therapy			4	15	19	*p* value^*^
Upgrade Ab therapy			9	8	17	
		Tot.	13	23	36	0.0467

Logistic regression of upgrade of antibiotic therapy				
Variable	Odds ratio	Standard error	95% conf. interval	*p* value
US grade ∆^¥^ ≥ 1	0.0801	0.0843	(0.0102–0.6304)	0.016
∆WBC**	1.0572	0.0688	(0.9307–1.2009)	0.392
∆CRP***	0.9883	0.0063	(0.9759–1.0008)	0.066
Age	1.1273	0.1171	(0.9196–1.3820)	0.249
Sex	2.0922	1.9665	(0.3315–13.2024)	0.432

^¥^US Grade ∆: difference between USINCHILD score at admission and after 48 h

*Fisher's exact test

**∆WBC: the difference between WBC evaluated after 48 h of follow-up and WBC at admission

***∆CRP: the difference between CRP evaluated after 48 h of follow-up and CRP at admission

The presence of an increase of at least 1 grade of USINCHILD score—ΔUS grade ≥ 1—was associated with the upgrade of empiric antibiotic therapy (logistic regression, *p* value: 0.016). The area under ROC curve of the change of USINCHILD was 0.87.

The evidence of USINCHILD score ≥ 1 was linearly associated with a statistically significant increase of defervescence time (*p* value: 0.036), as reported in Table 5.

**Table 5:** USINCHILD as predictor of resolution of fever*

Predictor variables	Haz. ratio	Standard error	95% conf. interval	*p* value
US Grade ∆^¥^ ≥ 1	2.3760	0.98	(1.0565–5.3436)	0.036
Age	1.0066	0.0425	(0.9266–1.0935)	0.876
Sex	0.8199	0.2914	(0.4086–1.6456)	0.576

*Defervescence time, expressed in hours, was chosen as expression of resolution of clinical signs

^¥^US Grade ∆: difference between USINCHILD score after 48hours and at admission. Cox regression analysys of defervescence time.

**Fig. 2:**
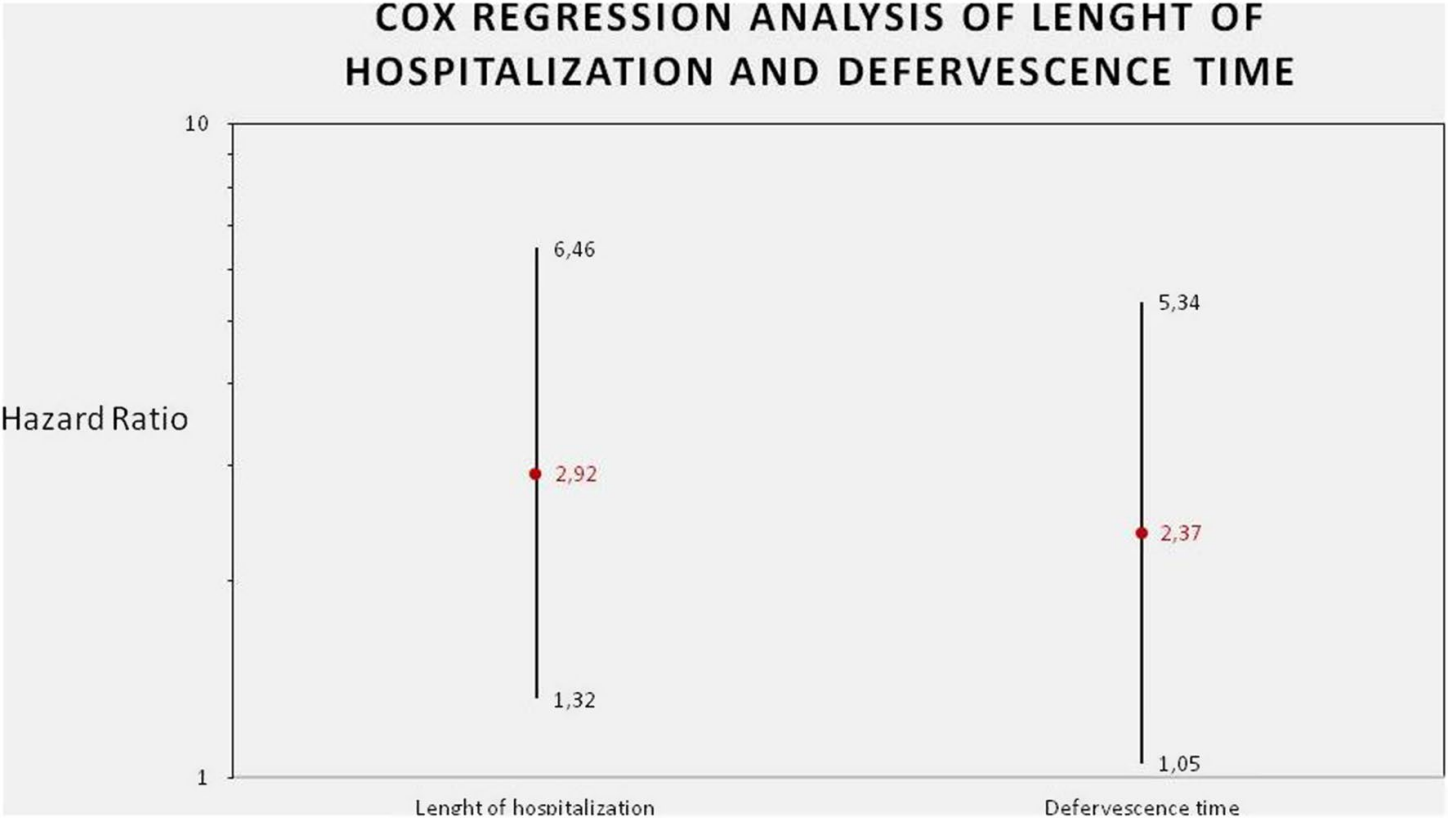
Cox regression analysis of lenght of hospitalization and defervescence time

Finally, the increase of at least 1 grade of the ultrasonographic score after 48 h was predictive of the length of hospitalization (*p* value: 0.008) (Table 6) (Fig. 2). In particular, the presence of Δ US grade ≥ 1 after 48 h was associated with a reduction of 7 days of hospitalization.

**Table 6:** USINCHILD as predictor of length of hospitalization

Predictor variables	Haz. ratio	Standard error	95% conf. interval	*p* value
US grade Δ^¥^ ≥ 1	2.9241	1.1831	(1.3231–6.4623)	0.008
Age	0.8982	0.0436	(0.8167–0.9879)	0.027
Sex	0.6895	0.2482	(0.3405–1.3962)	0.302

^¥^US Grade ∆: difference between USINCHILD score after 48hours and at admission. Cox regression analysys of lenght of hospitalization expressed as days.

## Discussion

Our study confirms that the evaluation of the characteristics of air bronchogram, a typical ultrasonographic finding of lung consolidation due to pneumonia, has an important impact in the management of pediatric CAP.

The first description of this ultrasonographic sign of alveolar consolidation, useful to differentiate pneumoniae from resorptive atelectasis, was reported by Lichtenstein et al. [17].

More recent studies confirmed and detailed the initial finding reported by Lichtenstein on dynamic air bronchogram as a sign ruling out atelectasis. The “linear arborescent” shape has then been identified as specific for pneumonia (either community-acquired [36] and ventilator-associated [38]). Moreover, both these studies proposed to use the air bronchogram to monitor pneumonia recovery [36, 37].

Recently, the Point of Care Ultrasound (POCUS) Working Group of the European Society of Paediatric and Neonatal Intensive Care (ESPNIC) provided evidence-based clinical guidelines for the use of POCUS in critically ill neonates and children [38]. The authors highlighted the diagnostic applications of LUS to detect pneumonia in neonates and children, remarking as typical ultrasonographic signs the presence of consolidations, dynamic air bronchograms, vertical artifacts (B lines), and pleural effusion. Moreover, it was recognized higher diagnostic accuracy of LUS compared with chest X-rays for the diagnosis of pneumonia [16].

To our knowledge, USINCHILD score is the first application of the prognostic role of ultrasonographic air bronchogram in the management of CAP in children.

In fact, the change of USINCHILD score is significantly related to the rate of complicated respiratory infections. Patients without a change of US score of at least 1 grade after 48 h are at risk of complicated CAP. Moreover, our data suggest that the presence of USINCHILD score after 48 h ≤ 1 is associated with an increased risk of complicated CAP.

Many lung ultrasound scores (LUS) are currently used to perform a qualitative assessment of lung aeration and guide respiratory care in lung disorders, because this is strongly recommended (level of evidence A) by current guidelines [40].

Raimondi et al. [40, 41] described the usefulness of LUS in predicting neonatal intensive care unit (NICU) admission or need for intubation, using only 3 simple LUS patterns.

In 2015, Brat et al. studied the diagnostic accuracy of a neonatal adapted LUS score to evaluate oxygenation and need for surfactant administration. The authors showed a good reliability to predict surfactant administration in preterm babies with a gestational age (GA) less than 34 weeks under continuous positive airway pressure [42].

In 2016, Rodríguez-Fanjul et al. evaluated the usefulness of LUS for prediction of the need for respiratory support in newborns older than 32 weeks showing good correlation with the need for mechanical ventilation and long respiratory support [43].

More recently, De Martino et al. explored the diagnostic accuracy of LUS in predicting the need for surfactant treatment and re-treatment in extremely preterm neonates with respiratory distress syndrome on CPAP [44].

As concern the possible association of USINCHILD score with the upgrade of antibiotic therapy not guided by microbiological examinations, our results show a significant correlation.

These results are concordant with the current guidelines for the management of pediatric CAP. In fact, the initial antibiotic treatment of CAP is empiric, because the pathogen is rarely known at the time of diagnosis. Children receiving adequate therapy should demonstrate clinical and also laboratory signs of improvement within 48–72 h. For children whose condition deteriorates after admission and initiation of antimicrobial therapy or who show no improvement within 48–72 h, it is often required an upgrade in the antibiotic therapy and further investigation to identify whether the original pathogen persists, whether it has developed resistance to the agent used, or whether there is a new secondary infecting agent.

The third outcome was the prognostic role of USINCHILD score on the time of resolution of clinical signs. We chose the fever as the most relevant clinical sign, because it was present in almost all patients at admission. Results show a strong association between the time to resolution of fever and the USINCHILD score after 48 h from admission.

Finally, our study demonstrates a clinically significant association of USINCHILD score with the length of hospitalization. Children with a difference of the grade equal or greater than 1 could present until 7 days of hospitalization less than patients without improvement of echographic score.

Our study has some limitations. First of all, the number of enrolled patients is limited. The number of patients enrolled is influenced by: (1) missing ultrasound data, (2) ultrasound data considered of not high quality, and (3) summer period within the time interval considered; second, the exploratory nature of the analysis. Nevertheless, the lack of previously published studies exploring the prognostic role of air bronchogram sign in pediatric field led us to preventively define a sample size of at least 30 patients in 1 year.

## Conclusions

In conclusion, our results demonstrate a prognostic role of the evaluation of the characteristics and the change of the air bronchogram in the management of CAP in children. Ultrasound examination at admission and after 48 h could give important information regarding the possible evolution toward a complicated respiratory infection.

Moreover, it may also help to identify children with favorable clinical course in terms of resolution of the clinical signs and length of hospitalization allowing to optimize the management of inpatient.

This study highlights that chest US is an excellent clinical tool not only for the diagnosis but also for the management of pediatric CAP.

## Electronic supplementary material

Below is the link to the electronic supplementary material.

Supplementary material 1 (avi 4472 kb)

Supplementary material 2 (avi 4656 kb)

Supplementary material 3 (avi 3132 kb)

Supplementary material 4 (avi 6477 kb)

Supplementary material 5 (avi 2693 kb)

Supplementary material 6 (avi 2847 kb)

## Data Availability

Deidentified individual participant data will not be made available.

## Abbreviations

CAP: Community-acquired pneumonia
US: Ultrasonographic
BTS: British Thoracic Society
CXR: Chest radiograph
NPS: Nasopharyngeal swab specimens
CRP: C-reactive protein
SD: Standard deviation
WBC: White blood cells
ΔUS: Change of USINCHILD score
